# Supplementary figures and images for: Induction of Immunological Antitumor Effects by the Combination of Adenovirus-Mediated Gene Transfer of B7-1 and Anti-Programmed Cell Death-1 Antibody in a Murine Squamous Cell Carcinoma Model
Source: Cancers (Basel). 2024 Mar 30;16(7):1359. doi: 10.3390/cancers16071359 (PMC11010972; doi:10.3390/cancers16071359)

# Supplementary Figure S1

Control

aPD-1

B7

aPD-1 + B7

Nuclei  
CD45

Nuclei  
CD3

Nuclei  
CD8

Nuclei  
FoxP3

Nuclei  
Ki67

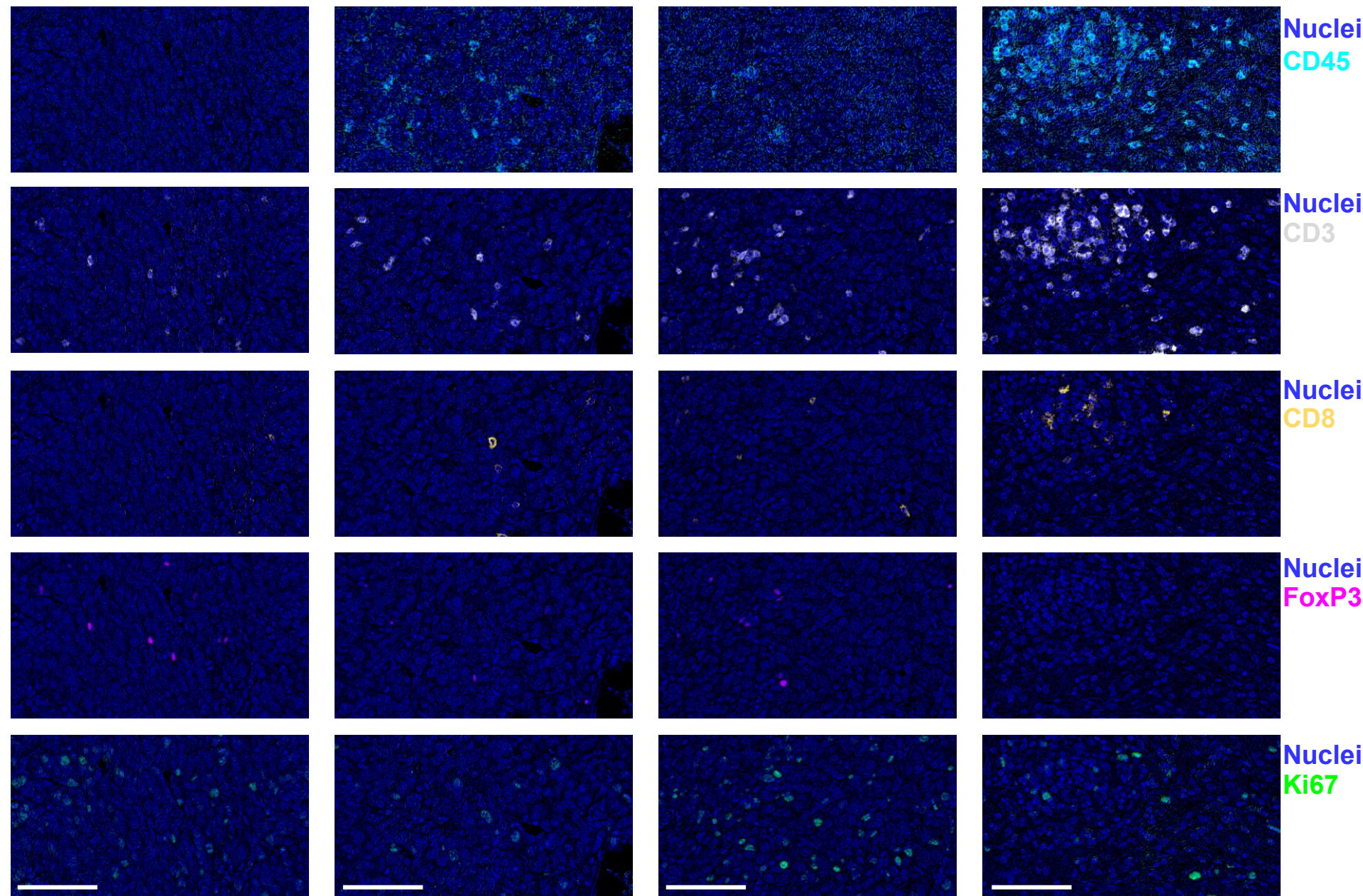

Supplement: Supplementary file 1 [file cancers-16-01359-s001.zip › cancers-2901822-supplementary.pdf]
